# Three new species of *Cucullanus* (Nematoda: Cucullanidae) from marine fishes off New Caledonia, with a key to species of *Cucullanus* from Anguilliformes

**DOI:** 10.1051/parasite/2018050

**Published:** 2018-09-20

**Authors:** František Moravec, Jean-Lou Justine

**Affiliations:** 1 Institute of Parasitology, Biology Centre of the Czech Academy of Sciences Branišovská 31 370 05 České Budějovice Czech Republic; 2 Institut Systématique, Évolution, Biodiversité (ISYEB), Muséum national d’Histoire naturelle, CNRS, Sorbonne Université, EPHE CP 51 57 rue Cuvier 75005 Paris France

**Keywords:** Parasitic nematode, taxonomy, Seuratoidea, Osteichthyes, *Conger*, *Gymnothorax*, *Dentex*, South Pacific

## Abstract

Based on light and scanning electron microscopical studies of nematode specimens from the digestive tract of some rarely collected anguilliform and perciform fishes off New Caledonia, three new species of *Cucullanus* Müller, 1777 (Cucullanidae) are described: *C*. *austropacificus* n. sp. from the longfin African conger *Conger cinereus* (Congridae), *C*. *gymnothoracis* n. sp. from the lipspot moray *Gymnothorax chilospilus* (Muraenidae), and *C*. *incognitus* n. sp. from the seabream *Dentex fourmanoiri* (Sparidae). *Cucullanus austropacificus* n. sp. is characterized by the presence of cervical alae, ventral sucker, alate spicules 1.30–1.65 mm long, conspicuous outgrowths of the anterior and posterior cloacal lips and by elongate-oval eggs measuring 89–108 × 48–57 μm; *C*. *gymnothoracis* n. sp. is similar to the foregoing species, but differs from it in the absence of cervical alae and the posterior cloacal outgrowth, in the shape and size of the anterior cloacal outgrowth and somewhat shorter spicules 1.12 mm long; *C*. *incognitus* n. sp. (based on female morphology) differs from other congeneric species parasitic in the Sparidae mainly in possessing cervical alae, the postequatorial vulva, phasmids situated at the mid-length of the tail and in the size of the eggs (75–84 × 45–66 μm). A key to species of *Cucullanus* parasitizing anguilliform fishes is provided.

## Abstract (fr)

Sur la base d’une étude en microscopie photonique et électronique à balayage de spécimens de nématodes provenant du tube digestif de quelques poissons anguilliformes et perciformes rarement pêchés en Nouvelle-Calédonie, trois nouvelles espèces de *Cucullanus* Müller, 1777 (Cucullanidae) sont décrites : *C. austropacificus* n. sp. de *Conger cinereus* (Congridae), *C. gymnothoracis* n. sp. de *Gymnothorax chilospilus* (Muraenidae) et *C. incognitus* n. sp. de *Dentex fourmanoiri* (Sparidae). *Cucullanus austropacificus* n. sp. est caractérisé par la présence d’ailes cervicales, une ventouse ventrale, des spicules ailés de 1,30-1,65 mm de long, des excroissances remarquables des lèvres cloacales antérieure et postérieure et d’oeufs ovales et allongés mesurant 89-108 × 48-57 μm. *C. gymnothoracis* n. sp. est semblable à l’espèce précédente, mais en diffère en l’absence d’ailes cervicales et d’excroissance cloacale postérieure, par la forme et la taille de l’excroissance cloacale antérieure et par des spicules un peu plus courts de 1,12 mm. *C. incognitus* n. sp. (basé sur la morphologie de la femelle) diffère des autres espèces congénères parasites chez les Sparidae principalement par la possession d’ailes cervicales, une vulve postéquatoriale, des phasmides situés à mi-longueur de la queue et par la taille des oeufs (75–84 × 45–66 μm). Une clé des espèces de *Cucullanus* parasitant les poissons anguilliformes est fournie.

The nematode genus *Cucullanus* Müller, 1777 (Cucullanidae) contains a large number of species parasitizing freshwater, brackish-water or marine fishes around the world; more rarely they are found in aquatic turtles [15, 27, 35]. Because of their rather uniform morphology and the inadequate descriptions of many nominal species, it is practically impossible to make a detailed comparison between all of them. Consequently, some authors prefer to deal with these parasites according to their host groups [12, 30, 35] or their zoogeographical region [9, 26, 44].

Only the following three nominal species of *Cucullanus*, all parasites of marine fishes, have been recorded from off New Caledonia: *C*. *bourdini* Petter & Le Bel, 1992 from *Aprion virescens* Valenciennes, *Lutjanus gibbus* (Forsskål), *Pristipomoides auricilla* (Jordan, Evermann & Tanaka) and *P*. *filamentosus* (Valenciennes) (all Lutjanidae); *C*. *bulbosus* (Lane, 1916) from *Carangoides fulvoguttatus* (Forsskål) (Carangidae); and *C*. *epinepheli* Moravec & Justine, 2017 from *Epinephelus chlorostigma* (Valenciennes) (Serranidae) [22–24, 36].

Parasitological examinations of some rarely collected marine fishes off New Caledonia conducted between 2009 and 2011 yielded, among other helminths, nematodes referable to *Cucullanus* from the digestive tract of *Conger cinereus* Rüppell (Congridae, Anguilliformes), *Gymnothorax chilospilus* Bleeker (Muraenidae, Anguilliformes), and *Dentex fourmanoiri* Akazaki & Séret (Sparidae, Perciformes). These proved to represent three morphologically different, previously unknown species of *Cucullanus*, which are described herein.

Whereas *Co*. *cinereus* and *G*. *chilospilus* are tropical, reef-associated fishes widespread in the Indo-Pacific region, *D*. *fourmanoiri* is a rare, deep-water fish with a limited distribution in the Southwest Pacific, occurring near the Chesterfield Islands and New Caledonia [11].

## Materials and methods

Fish were caught off New Caledonia by various, and sometimes unusual, means. The seabream *Dentex fourmanoiri* was caught by line; the conger *Conger cinereus* was taken in a cage baited for the collection of *Nautilus*; and the moray *Gymnothorax chilospilus* was obtained from a New Caledonian sea krait, *Laticauda saintgironsi* Cogger & Heatwole, collected on a small islet, Ilôt Amédée, off Nouméa, New Caledonia. As this host is an emblematic protected species, an indirect sampling method without any effect on survival was used [5]: a gentle massage of the sea krait abdomen provided the stomach content by regurgitation, and the regurgitated contents included the moray eel. Parasites were obtained by a wash method [14]. The nematodes for morphological studies were fixed in hot 4% formalin or 70% ethanol. For light microscopical examination (LM), they were cleared with glycerine. Drawings were made with the aid of a Zeiss microscope drawing attachment. Specimens used for scanning electron microscopical examination (SEM) were postfixed in 1% osmium tetroxide (in phosphate buffer), dehydrated through a graded acetone series, critical-point-dried and sputter-coated with gold; they were examined using a JEOL JSM-7401F scanning electron microscope at an accelerating voltage of 4 kV (GB low mode). All measurements are in micrometres, unless otherwise indicated. The fish nomenclature adopted follows FishBase [11].

## Results

Cucullanidae Cobbold, 1864

### *Cucullanus austropacificus* n. sp. Figures 1–3

urn:lsid:zoobank.org:act:4F1D1180-8FE8-498F-A38C-2C63A4C9A36E

**Figure 1.**
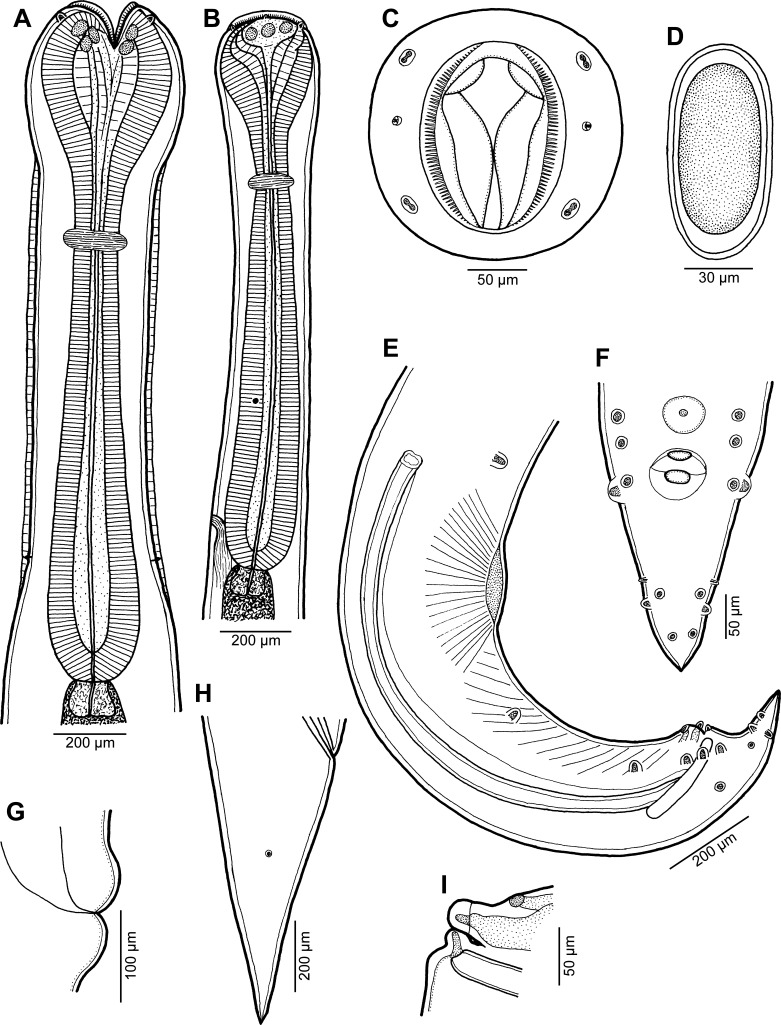
*Cucullanus austropacificus* n. sp. from *Conger cinereus*. (A) Anterior end of large male (body length 24 mm), dorsoventral view; (B) anterior end of small male (body length 17 mm), lateral view; (C) cephalic end, apical view; (D) fully developed egg; (E) posterior end of male, lateral view; (F) tail of male, ventral view; (G) vulva, lateral view; (H) tail of female, lateral view; (I) region of cloaca, lateral view.

Type host: Longfin African conger *Conger cinereus* Rüppell (Congridae, Anguilliformes).

Site of infection: Digestive tract.

Type locality: Deep sea, 400 m depth, near Passe de Dumbéa, off Nouméa, New Caledonia (collected 3 July 2009).

Prevalence and intensity: 1 fish infected/1 fish examined; 12 nematodes.

Deposition of type specimens: Muséum national d’Histoire naturelle, Paris, France (male holotype, female allotype and 3 paratypes [2 males and 1 female], MNHN JNC 2993); Helminthological Collection, Institute of Parasitology, Biology Centre of the Czech Academy of Sciences, České Budějovice, Czech Republic (3 paratypes [2 males and 1 female], Cat. No. N–1167).

Etymology: The specific name of this nematode is a Latin adjective composed of the words *australis* (= southern) and *Pacificus* (= Pacific), which relates to the region of the occurrence of this parasite, that is South Pacific.

#### Description

**Figure 2.**
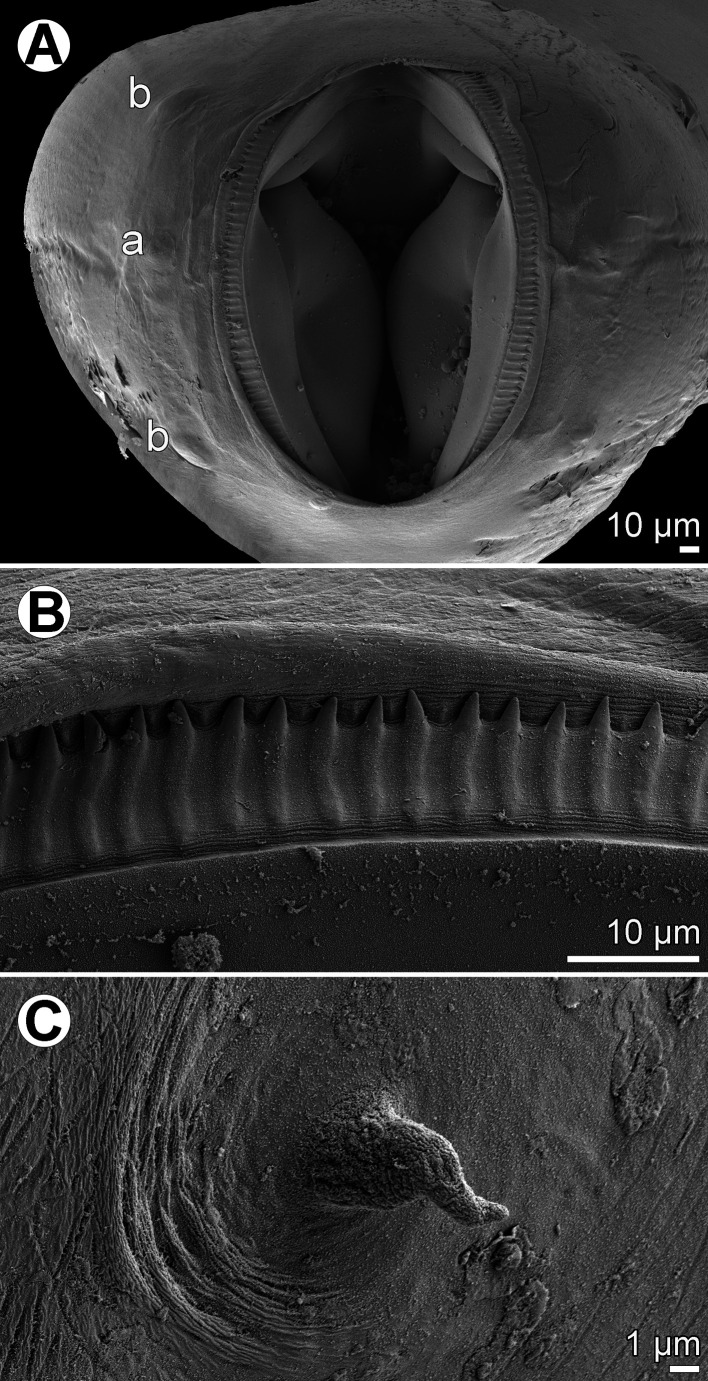
*Cucullanus austropacificus* n. sp., scanning electron micrographs. (A) Cephalic end, apical view; (B) cephalic teeth; (C) deirid. (a) amphid; (b) cephalic double papilla.

**Figure 3.**
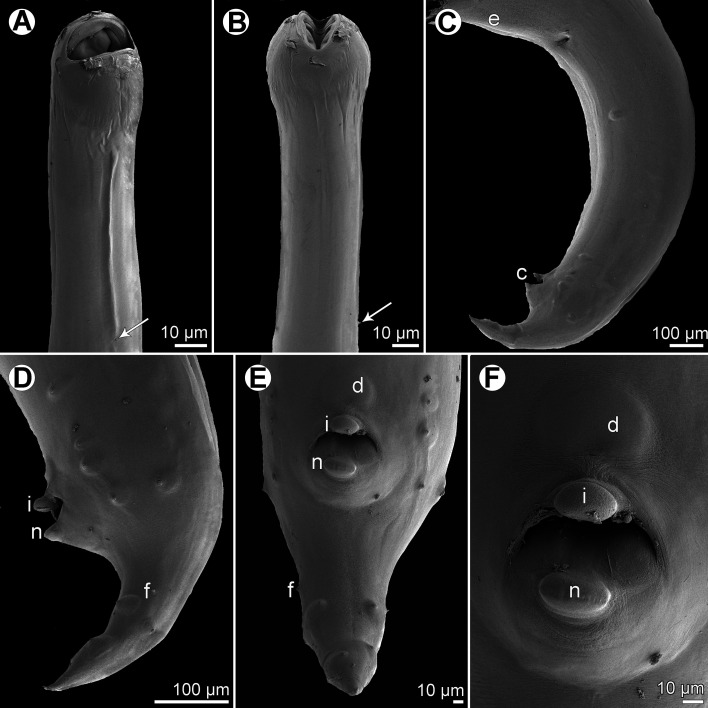
*Cucullanus austropacificus* n. sp., scanning electron micrographs. (A, B) Anterior end of body, sublateral and dorsoventral views, respectively (arrows indicate deirids; note presence of cervical alae); (C) posterior end of male, lateral view; (D, E) tail of male, lateral and ventral views, respectively; (F) region of cloaca, ventral view (higher magnification). (c) cloaca; (d) median precloacal papilla-like formation; (e) ventral sucker; (f) phasmid; (i) median outgrowth of anterior cloacal lip; (n) median outgrowth of posterior cloacal lip.

*General*: Medium-sized nematodes. Body whitish, elongate, somewhat narrowed in region between posterior end of pseudobuccal capsule and posterior end of oesophagus (Figs. 1A and 1B). Narrow lateral cervical alae present, beginning approximately at level of posterior end of pseudobuccal capsule and extending posteriorly to short distance anterior to posterior end of oesophagus (Figs. 1A, 3A and 3B). Cephalic end slightly asymmetrical in lateral view (Figs. 1B, 1C and 2A). Oral aperture dorsoventrally elongate, surrounded by raised narrow membranous ala (collarette) supported by row of *c.* 100 minute basal teeth (Figs. 1C, 2A, and 2B). Four submedian cephalic double papillae and pair of lateral amphids present (Figs. 1C and 2A). Oesophagus muscular, expanded at anterior end to form bulbous pseudobuccal capsule (oesophastome); posterior part of oesophagus also expanded, somewhat narrower than oesophastome in lateral view (Figs. 1A and 1B). Oesophagus opens into intestine through large valve. Nerve ring encircles oesophagus at distance representing 29%–35% of oesophageal length. Deirids small, situated in posterior half of distance between nerve ring and posterior end of oesophagus (Figs. 1A, 1B, 2C, 3A, 3B). Postdeirids not found. Excretory pore in region of oesophago-intestinal junction (Fig. 1B). Tail of both sexes conical, sharply pointed at tip.

*Male* (7 specimens; measurements of holotype in parentheses): Length of body 15.80–26.15 (26.15) mm, maximum width 422–680 (680); width at level of oesophastome 286–490 (490), at middle of oesophagus 218–408 (408). Maximum width of cervical alae 18–27 (24). Length of entire oesophagus 1.59–2.31 (2.31) mm, representing 9–11 (9)% of whole body length; length of oesophastome 340–530 (503), its width 272–408 (394); minimum width of oesophagus 95–150 (150); maximum width of posterior part of oesophagus 217–299 (299). Distance of nerve ring from anterior extremity 503–789 (789), representing 29–35 (34)% of oesophageal length. Deirids and excretory pore 1.02–2.12 (2.12) mm and 1.32–2.57 (2.01) mm, respectively, from anterior end of body. Posterior end of body curved ventrally. Ventral sucker and ventral precloacal oblique muscle bands present (Figs. 1E, 3C). Cloacal region somewhat elevated. Large median papilla-like formation present anterior to cloacal opening, being accompanied by conspicuously elevated transversely-oval median outgrowth adherent to inner rim of anterior cloacal lip; posterior cloacal lip with median, transversely-oval, conspicuously elevated outgrowth at tip (Figs. 1E, 1F, 1I, 3C–3F). Spicules equal, 1.30–1.65 (1.59) mm long, representing 5–10 (6)% of body length. Gubernaculum well sclerotized, rod-like in lateral view, 225–291 (291) long (Fig. 1E). Caudal papillae 11 pairs: 5 pairs of subventral preanal papillae, 2 pairs of adanal papillae (1 subventral and 1 lateral) and 4 pairs of postanal papillae (2 subventral, 1 lateral and 1 dorsolateral); subventral pairs of postanals in second half of tail; postanal pair of laterals (representing phasmids) slightly anterior to level of first subventral pair; papillae of dorsolateral postanal pair slightly anterior to level of last pair of subventrals (Figs. 1E, 1F, 3C–3E). Length of tail 219–462 (462) (Figs. 1E, 1F, 3C–3E).

*Female* (5 ovigerous specimens; measurements of allotype in parentheses): Length of body 26.43–34.61 (34.61) mm, maximum width 680–734 (734); width at level of oesophastome 476–558 (558), at middle of oesophagus 408–422 (408). Maximum width of cervical alae 24–33 (33). Length of entire oesophagus 2.42–2.58 (2.58), representing 7–9 (7)% of whole body length; length of oesophastome 544–625 (612), its width 422–462 (462); minimum width of oesophagus 109–163 (109); maximum width of posterior part of oesophagus 272–326 (326). Distance of nerve ring from anterior extremity 789–857 (857), representing 32–33 (33)% of oesophageal length. Deirids and excretory pore 1.84–1.97 (1.96) and 2.34–2.77 (2.77) mm, respectively, from anterior end of body. Vulva postequatorial, 15.89–20.84 (20.84) mm from anterior extremity, at 60–63 (60)% of body length; vulval lips slightly elevated (Fig. 1G). Vagina directed anteriorly from vulva. Uteri opposed. Fully developed eggs elongate-oval, thin-walled, size 84–108 × 48–57 (93–99 × 54–57), with uncleaved contents (Fig. 1D). Length of tail 666–857 (694); phasmids situated approximately at its middle (Fig. 1H).

#### Remarks

To date, the following 12 species of *Cucullanus* are known to occur in anguilliform fishes: *C*. *anguillae* Wang & Ling, 1975 from *Anguilla japonica* (Temminck & Schlegel) in China [45]; *C*. *australiensis* Baylis, 1927 (syn. *C*. *faliexae* Morand & Rigby, 1998) from *Gymnothorax* cf. *pictus* (Ahl) and *G*. *javanicus* (Bleeker) from off Australia and French Polynesia, respectively [2, 18]; *C*. *egyptae* Abdel-Ghaffar, Bashtar, Abdel-Gaber, Morsy, Mehlhorn, Al Quraishy & Mohammed, 2014 [*species inquirenda*; 31] from *Anguilla anguilla* (Linnaeus) in Egypt [1]; *C*. *hainanensis* Xu, Zhang & Li, 2014 from *Muraenichthys gymnopterus* (Bleeker) in the South China Sea [41]; *C*. *hians* (Dujardin, 1845) [syn. *C*. *praecinctus* (Dujardin, 1845)] mainly from *Conger conger* (Linnaeus) off the Atlantic coasts of Europe and Africa [8, 10, 37]; *C*. *muraenesocis* Yamaguti, 1961 from *Muraenesox cinereus* (Forsskål) off Japan [47]; *C*. *murenophidis* Campana-Rouget, 1957 from *Muraena robusta* Osório off the Atlantic coast of Africa [7]; *C*. *oceaniensis* Moravec, Sasal, Würtz & Taraschewski, 2005 from *Anguilla marmorata* Quoy & Gaimard and *Anguilla* cf. *obscura* Günther in Oceania [30]; *C*. *pedroi* Timi & Lanfranchi, 2006 from *Conger orbignianus* Valenciennes off the Atlantic coast of Argentina and Brazil [40, 44]; *C*. *robustus* Yamaguti, 1935 (syn. *C*. *filiformis* Yamaguti, 1935) from *Conger myriaster* (Brevoort) from off Japan and the Korean Peninsula [32, 46]; *C*. *truttae* Fabricius, 1794 from *Anguilla anguilla* (but mostly parasitic in freshwater salmonids and cyclostomes) in Europe [20, 21]; and *C*. *wangi* Xu, Zhang & Li, 2014 (syn. *Indocucullanus muraenesocis* Yin & Zhang, 1983) from *Muraenesox cinereus* off China [41, 48].

*Cucullanus austropacificus* n. sp. differs from all of the above-mentioned species, except for *C*. *truttae*, in the presence of cervical alae, but also in some other morphological features. By the structure of the cloacal region, the new species is most similar to *C*. *pedroi* parasitizing congeneric fish host in the western Atlantic Ocean, but differs from it in the shape (more elongate in *C*. *pedroi*) of the oesophastome, its anterior cloacal outgrowth is smaller than the posterior outgrowth (*vs*. anterior outgrowth larger than the posterior one), the lower posterior part of the posterior cloacal lip is without denticulations (*vs*. denticulations present) and the sixth pair of subventral papillae is situated posterior to the cloaca (*vs.* at the level of the cloaca) (see Figs. 60–62 in Vieira *et al*. [44]). The distinction of *C*. *austropacificus* n. sp. from other congeners parasitizing anguilliform fishes is more apparent from the key at the end of the Discussion in this article.

In the same individual conger, we also collected digeneans, including *Acaenodera nautili* Bray & Justine, 2011, and larvae of the trypanorhynch cestode *Microbothriorhynchus coelorhynchi* Yamaguti, 1952 [3, 4].

### *Cucullanus gymnothoracis* n. sp. Figures 4–6

urn:lsid:zoobank.org:act:D5E65F49-66D2-466C-BC48-5EC0CC88E1F9

**Figure 4.**
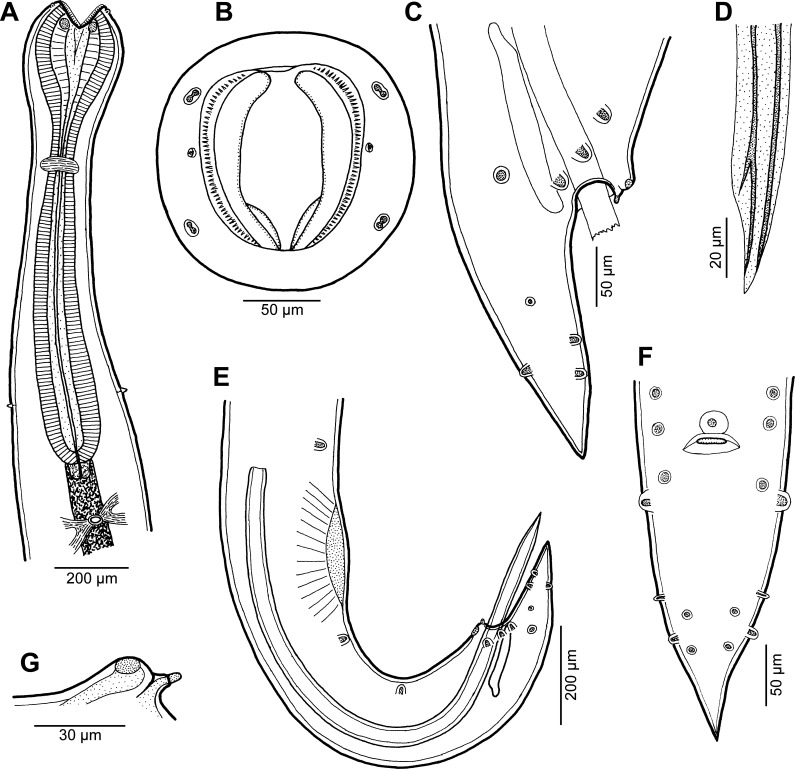
*Cucullanus gymnothoracis* n. sp. from *Gymnothorax chilospilus*, male. (A) Anterior end of body, ventral view; (B) cephalic end, apical view; (C) tail, lateral view; (D) distal end of spicule, lateral view; (E) posterior end of body, lateral view; (F) tail, ventral view; (G) median precloacal papilla-like formation and outgrowth of anterior cloacal lip, lateral view.

Type host: Lipspot moray *Gymnothorax chilospilus* Bleeker (Muraenidae, Anguilliformes), obtained from the stomach content of a male New Caledonian sea krait, *Laticauda saintgironsi* Cogger & Heatwole, 2006 (see Materials and methods section).

Site of infection: Digestive tract.

Type locality: Near Ilôt Amédée, off Nouméa, New Caledonia (collected 13 February 2011) (parasitological number MNHN JNB 004).

Prevalence and intensity: 1 fish infected/5 fish examined; 1 nematode.

Deposition of type specimen: Helminthological Collection, Institute of Parasitology, Biology Centre of the Czech Academy of Sciences, České Budějovice, Czech Republic (male holotype mounted on SEM stub, Cat. No. N–1168).

Etymology: The specific name of this nematode relates to the genitive form of the generic name of the host.

#### Description

**Figure 5.**
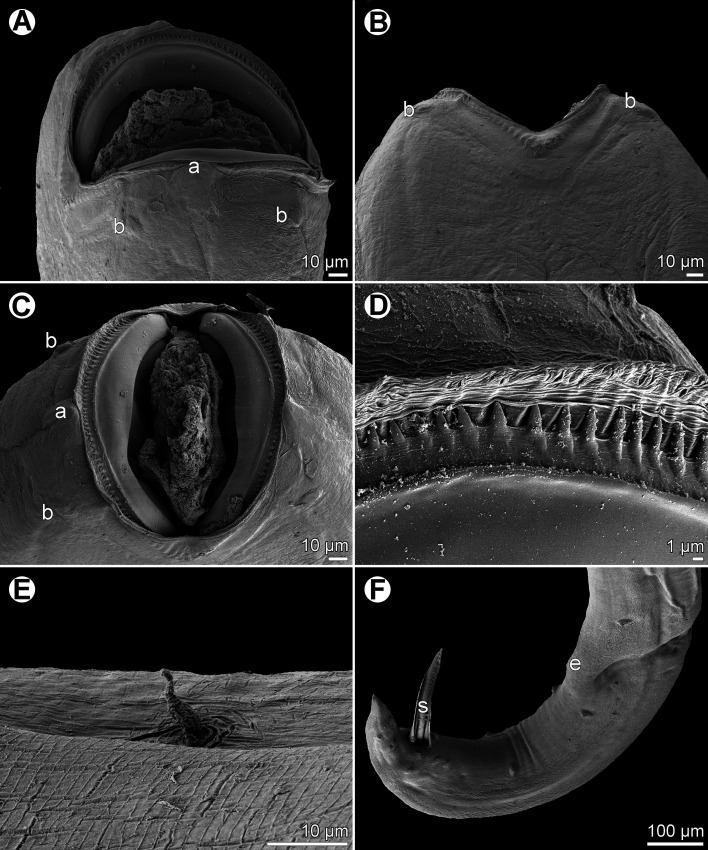
*Cucullanus gymnothoracis* n. sp., scanning electron micrographs of male. (A, B) Cephalic end, lateral and dorsoventral views, respectively; (C) same, apical view; (D) cephalic teeth; (E) deirid; (F) posterior end of body, lateral view. (a) amphid; (b) cephalic double papilla; (e) ventral sucker; (s) spicule.

**Figure 6.**
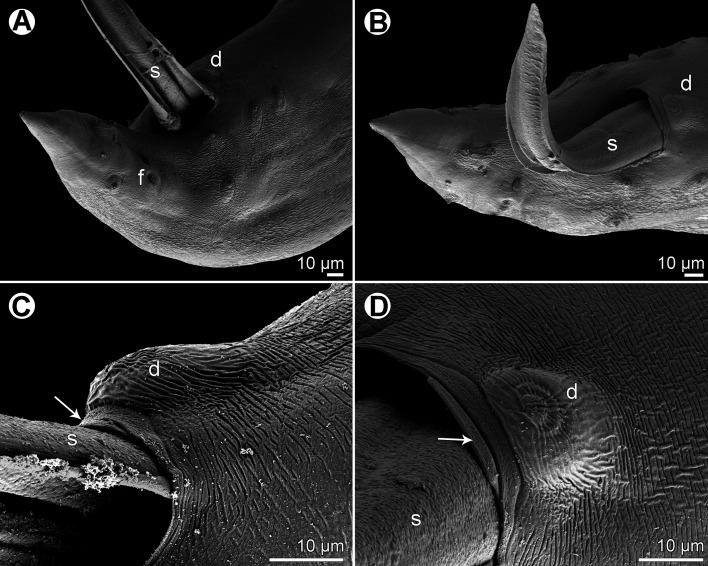
*Cucullanus gymnothoracis* n. sp., scanning electron micrographs of male. (A, B) Tail, lateral and ventral views, respectively; (C, D) precloacal region, lateral and ventral views, respectively (arrows indicate outgrowth of anterior cloacal lip). (d) median precloacal papilla-like formation; (f) phasmid; (s) spicule.

*Male* (1 specimen, holotype): Medium-sized nematode. Body whitish, elongate, somewhat narrowed in region between posterior end of pseudobuccal capsule and posterior end of oesophagus (Fig. 4A). Cuticle slightly transversely striated (Figs. 5E, 6C, 6D). Length of body 14.63 mm, maximum width 394; width at level of oesophastome 286, at middle of oesophagus 177. Lateral alae absent (Figs. 4A, 5E). Cephalic end somewhat asymmetrical in lateral view (Fig. 5A). Oral aperture dorsoventrally elongate, surrounded by raised narrow membranous ala (collarette) supported by row of *c.* 120 minute basal teeth (Figs. 4B, 5A, 5C, 5D). Four submedian cephalic double papillae and pair of lateral amphids present (Figs. 4B, 5A–5C). Oesophagus muscular, expanded at anterior end to form bulbous pseudobuccal capsule (oesophastome); posterior part of oesophagus also expanded, somewhat narrower than oesophastome in lateral view (Fig. 4A). Length of entire oesophagus 1.40 mm, representing 9.6% of whole body length; length of oesophastome 367, its width 258; minimum width of oesophagus 68; maximum width of posterior part of oesophagus 177. Oesophagus opens into intestine through large valve. Distance of nerve ring from anterior extremity 480, representing 35% of oesophageal length. Deirids small, situated short distance anterior to posterior end of oesophagus (Figs. 4A, 5E). Postdeirids not found. Excretory pore situated at short distance posterior to oesophago-intestinal junction (Fig. 4A). Deirids and excretory pore 1.16 mm and 1.48 mm, respectively, from anterior end of body. Posterior end of body curved ventrally. Ventral precloacal sucker present (Figs. 4E, 5F). Cloacal region not elevated. Large, somewhat elevated median papilla-like formation present anterior to cloacal opening, being accompanied by slightly elevated median transverse outgrowth adherent to inner rim of anterior cloacal lip (Figs. 4C, 4E–4G, 6A–6D). Spicules equal, alate, 1.12 mm long, with pointed posterior ends (Figs. 4D, 4E, 5F, 6B), representing 7.6% of body length. Gubernaculum well sclerotized, rod-like with narrow proximal part in lateral view, 201 long (Figs. 4C, 4E). Caudal papillae 11 pairs: 5 pairs of subventral preanal papillae, 2 pairs of adanal papillae (1 subventral and 1 lateral) and 4 pairs of postanal papillae (2 subventral, 1 lateral and 1 dorsolateral); subventral pairs of postanals in second half of tail; postanal pair of laterals (representing phasmids) slightly anterior to level of first subventral pair; papillae of dorsolateral postanal pair slightly anterior to level of last pair of subventrals (Figs. 4C, 4E, 4F, 5F, 6A, 6B). Tail conical, pointed, 245 long (Figs. 4C, 4E, 4F, 5F, 6A, 6B).

*Female*: Not known.

#### Remarks

The morphology and measurements of this nematode specimen, as well as the fact that it was collected from the congeneric fish host in the nearby region, show its similarity to *C*. *australiensis*, the species originally described by Baylis [2] from *Gymnothorax* cf. *pictus* off Australia. Later Morand & Rigby [18] established *C*. *faliexae* from *G*. *javanicus* in French Polynesia, but it was subsequently synonymized with *C*. *australiensis* [30]. *Cucullanus australiensis* has not yet been studied by SEM, so its detailed morphology remains unknown. Nevertheless, the present specimen differs markedly from *C*. *australiensis* in the considerably more posterior situation of deirids and the excretory pore and, therefore, this is considered to represent a separate species. Comparison of *C*. *gymnothoracis* n. sp. with other congeneric species is apparent from the key presented at the end of the Discussion in this article.

### *Cucullanus incognitus* n. sp. Figures 7, 8

urn:lsid:zoobank.org:act:C2C4E9A4-A750-4D94-961F-1B9AEA3B79AF

**Figure 7.**
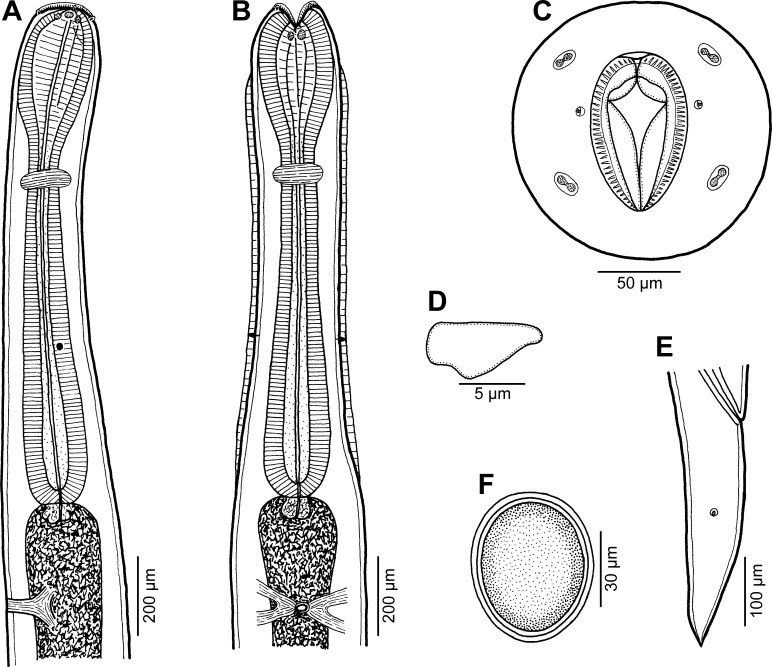
*Cucullanus incognitus* n. sp. from *Dentex fourmanoiri*, female. (A, B) Anterior end of body, lateral and ventral views; (C) cephalic end, apical view; (D) deirid, lateral view; (E) tail, lateral view; (F) egg.

**Figure 8.**
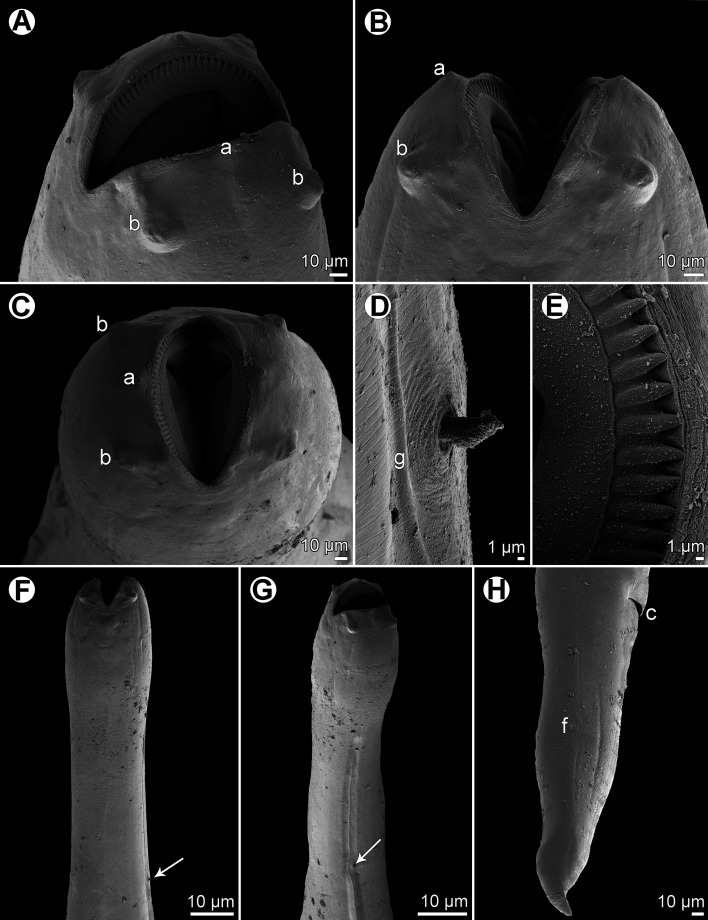
*Cucullanus incognitus* n. sp., scanning electron micrographs of female. (A, B) Cephalic end, sublateral and dorsoventral views, respectively; (C) same, apical view; (D) deirid and cervical ala, sublateral view; (E) cephalic teeth; (F, G) anterior end of body, dorsoventral and sublateral views, respectively (arrows indicate deirids; note cervical alae); (H) tail, lateral view. (a) amphid; (b) cephalic double papilla; (c) anus; (f) phasmid; (g) cervical ala.

Type host: *Dentex fourmanoiri* Akazaki et Séret (Sparidae, Perciformes) (total body length 242 mm, weight 349 g).

Site of infection: Digestive tract.

Type locality: Deep sea, external slope of the barrier reef, off Récif Toombo, near Nouméa, New Caledonia (22º34′841S, 166º27′612E, depth 200–350 m) (collected 2 July 2009).

Prevalence and intensity: 1 fish infected/1 fish examined; 2 specimens.

Deposition of type specimens: Muséum national d’Histoire naturelle, Paris, France (female holotype, MNHN JNC 2992); Helminthological Collection, Institute of Parasitology, Biology Centre of the Czech Academy of Sciences, České Budějovice, Czech Republic (female paratype mounted on SEM stub, Cat. No. N–1169).

Etymology: The specific name *incognitus* (= unknown) is a Latin adjective and relates to the fact that this nematode species was previously unknown.

#### Description

*Female* (2 ovigerous specimens; holotype; measurements of paratype in parentheses): Medium-sized nematodes. Body whitish, elongate, slightly narrowed in region between posterior end of pseudobuccal capsule and posterior end of oesophagus (Figs. 7A, 7B, 8F, 8G); length of body 15.14 (13.38) mm, maximum width 354 (354); body length at level of oesophastome 272 (245), at middle of oesophagus 258 (231). Narrow lateral cervical alae present, 15 (15) wide, commencing approximately at level of mid-length of pseudobuccal capsule and extending posteriorly to short distance anterior to posterior end of oesophagus (Figs. 7B, 8D, 8F, 8G). Cephalic end slightly asymmetrical in lateral view (Figs. 7A, 8A). Oral aperture dorsoventrally elongate, surrounded by raised narrow membranous ala (collarette) supported by row of *c.* 90 minute basal teeth (Figs. 7C, 8A–8C, 8E). Four submedian cephalic double papillae and pair of lateral amphids present (Figs. 7C, 8A–8C, 8F, 8G). Oesophagus muscular, expanded at anterior end to form elongate pseudobuccal capsule (oesophastome), approximately twice as long as wide (Figs. 7A, 7B); length of oesophastome 394 (394), width 231 (204). Posterior part of oesophagus also expanded, somewhat narrower than oesophastome in lateral view (Figs. 7A, 7B); maximum width of posterior part of oesophagus 204 (177), minimum width of oesophagus in region of nerve ring 109 (82). Entire length of oesophagus including oesophastome 1.54 (1.40) mm, representing 10 (10)% of total body length. Oesophagus opens into intestine through large valve. Nerve ring encircles oesophagus at distance of 503 (490) from anterior extremity, representing 33 (35)% of oesophageal length. Deirids situated approximately at mid-way between nerve ring and posterior end of oesophagus, at 952 (938) from anterior end of body (Figs. 7A, 7B, 8D, 8F, 8G). Postdeirids not found. Excretory pore at short distance posterior to oesophago-intestinal junction (Fig. 7A, 7B), at 1.71 (1.61) mm from anterior extremity. Vulva situated 9.14 (8.16) mm from anterior end of body, i.e. at 60 (61)% of body length; vulval lips not elevated. Vagina directed anteriorly from vulva. Eggs oval, thin-shelled, size 75–84 × 45–66 (81–90 × 54–60) (Fig. 7F). Tail conical, 394 (394) long, sharply pointed at tip, provided with pair of lateral phasmids located approximately at its middle (Figs. 7E, 8H).

*Male*: Not known.

#### Remarks

Even though males of this new species are not known, *C*. *incognitus* n. sp. can be distinguished from the great majority of *Cucullanus* spp. by the presence of lateral cervical alae. Of the many species of *Cucullanus*, the presence of cervical alae, as found in *C*. *incognitus*, has hitherto been described only in *C*. *truttae*, a parasite mainly of freshwater salmonids (Salmonidae) in the Holarctic [21, 28], and in two recently established species parasitizing marine fishes in New Caledonian waters, i.e., *C*. *epinepheli* Moravec & Justine, 2017 parasitic in *Epinephelus chlorostigma* (Valenciennes) (Serranidae, Perciformes) and *C*. *austropacificus* n. sp. from *Conger cinereus* reported in the present paper. Rasheed [38] mentioned the presence of asymmetrical “cuticular expansions in the form of cephalic alae” in *C*. *theraponi* Rasheed, 1968 from “*Therapon*” (= *Terapon*?) sp. (Terapontidae, Perciformes) and *Hilsa* sp. (Clupeidae, Clupeiformes) from off Pakistan, but these formations are different from cervical alae.

*Cucullanus incognitus* n. sp. differs from *C*. *epinepheli* in that its posterior portion of the oesophagus is narrower (*vs*. markedly wider) than the anterior oesophastome, its deirids are situated more anteriorly in relation to the length of the oesophagus (at 68% *vs*. 77–86% of oesophagus length) and the hosts belong to different fish families (Sparidae *vs.* Serranidae). In contrast to *C*. *austropacificus*, the gravid females of the new species are much smaller (body length approximately 13–15 mm *vs.* 26–35 mm), their oesophastome is more elongate (approximately twice as long as wide *vs.* approximately as long as wide) and their eggs are smaller (75–84 × 45–66 μm *vs.* 84–108 × 48–57 μm) and of a different shape (oval *vs.* elongate-oval); hosts of these two species belong to different fish orders (Perciformes *vs.* Anguilliformes). Regarding *C*. *truttae*, it can be easily distinguished from *C*. *incognitus* by the conspicuously asymmetrical cephalic end and by the excretory pore located at the mid-distance between the nerve ring and the oesophago-intestinal junction (*vs.* excretory pore posterior to the oesophageal end).

To date, the only nominal species of *Cucullanus* previously described from seabreams (Sparidae) are *C*. *chrysophrydis* Gendre, 1928, parasitizing *Pagellus bogaraveo* (Brünnich) and *Sparus aurata* Linnaeus off the Atlantic coast of Africa [7, 13, 43], and *C*. *protrudens* Pereira, Vieira & Luque in Vieira *et al.*, 2015, a parasite of *Pagrus pagrus* (Linnaeus) from off the Atlantic coast of Brazil [44]. Both these species differ from *C*. *incognitus* n. sp. in the absence (*vs.* presence) of cervical alae and in the location of phasmids in the second half of the tail (*vs.* at mid-length of tail). In addition, the vulva of *C*. *protrudens* is preequatorial, at 41%–43% of the body length (*vs.* postequatorial in the new species, at 60%–61% of body length). Moreover, all these three nematode species parasitize hosts belonging to different genera (*Pagellus* Valenciennes, *Pagrus* Cuvier and *Sparus* Linnaeus *vs Dentex* Cuvier) and they occur in geographically very distant regions (*C*. *chrysophrydis* and *C*. *protrudens* in the Atlantic Ocean *vs C*. *incognitus* n. sp. in the Pacific Ocean).

An unidentified species of *Cucullanus*, *Cucullanus* sp. from *Pagrus* sp., was reported by Vassiliadès [43] in the list of helminth parasites of marine fishes off the coast of Senegal (Atlantic Ocean). *Cucullanus* sp. was also reported from *Pagrus auratus* (Forster) in the Pacific Ocean, New Zealand [39]. However, in this case, specimens of another cucullanid genus *Dichelyne* Jägerskiöld, 1902 were probably misidentified as *Cucullanus*, as indicated by the small body measurements and accompanying illustrations (the intestinal caecum in *Dichelyne* spp. is sometimes difficult to observe and was probably overlooked by the authors). This is also supported by the fact that *Cucullanellus* (= *Dichelyne*) *cnidoglanis* Johnston & Mawson, 1945 was reported from the same host species (*P*. *auratus*) in the same region (off New Zealand) [6]. Three species of the Sparidae, *Acanthopagrus schlegelii* (Bleeker), *Dentex* (reported as *Evynnis* Jordan & Thompson) *tumifrons* (Temminck & Schlegel) and *Pagrus major* (Temminck et Schlegel), were reported as hosts of *Dichelyne jialaris* Luo, Guo, Fang & Huang, 2004 from off China and Japan [16, 29].

The authors are aware of the fact that the description of *C*. *incognitus* n. sp. is based solely on female morphology, a procedure that cannot generally be recommended; however, in this case, the new species appears to be well established and, therefore, we consider it useful to give the species a name rather than to report it as *Cucullanus* sp. and to wait years until conspecific males are available; the host is extremely rarely collected.

It should be noted that the only host specimen (*D*. *fourmanoiri*) examined harboured, in addition to *C*. *incognitus* n. sp., two specimens of the cystidicolid nematode *Rasheedia heptacanthi* Moravec & Justine, 2018 in the digestive tract [25].

## Discussion

As mentioned above, the morphology of the numerous species of *Cucullanus* is rather uniform. Therefore, the separation of similar species based solely on morphological features studied by LM may be problematic, especially in the situation when some *Cucullanus* spp. have been insufficiently described. Nevertheless, some papers published during last two decades (e.g. [15, 17, 22–24, 27, 30, 32–34, 41, 42, 44]) have shown the importance of the use of SEM for the taxonomy of these nematodes, because some features are difficult to observe or are not visible at all under the LM.

This concerns, for example, the exact number and distribution of caudal papillae in the male or the situation of deirids and the excretory pore. One such feature is the presence of narrow lateral cervical alae, observable in dorsoventral view, which can be easily overlooked when using LM, but their presence can be confirmed by SEM. The presence/absence of cervical alae appears to be an important specific taxonomic feature in *Cucullanus*; as stated above, of many described species of *Cucullanus*, cervical alae have hitherto been reported only in *C*. *austropacificus* n. sp., *C*. *epinepheli*, *C*. *incognitus* n. sp. and *C*. *truttae*. According to experimental observations [19], cervical alae of third- and fourth-stage larvae of *C*. *truttae* are much wider than those of conspecific adults.

As revealed by SEM, taxonomically very important features are found in the structures of the cloacal region in the male. It seems that many species of *Cucullanus* possess a small, rounded median precloacal elevation, sometimes reported as the median precloacal papilla or the median precloacal organ; in fact, such an elevation usually bears a single minute papilla (e.g. in *C*. *gymnothoracis* n. sp., see Fig. 6D) or, less often, two minute papillae (*C*. *epinepheli*, *C*. *genypteri* Sardella, Navone & Timi, 1997) are visible on its surface [44], present paper. However, there are species of *Cucullanus* (e.g. *C*. *bulbosus*), in which the median precloacal elevation is lacking [22].

In species of *Cucullanus* possessing the median precloacal elevation, the elevation may be connected with the conspicuously large, flat posterior outgrowth of the anterior cloacal lip that covers the cloacal aperture (in *C*. *epinepheli*) or there is a median, ventrally oriented outgrowth protruding from the base of the anterior cloacal lip; this may be rounded and small (e.g. in *C*. *mycteropercae* Mejía-Madrid & Guillén-Hernández, 2011, *C*. *pseudopercis* Pereira, Vieira & Luque in Vieira et al., 2015 or *C*. *oceaniensis* Moravec, Sasal, Würtz & Taraschewski, 2005) or large (e.g. in *C*. *pedroi* or *C*. *austropacificus* n. sp., see Fig. 3F), or the outgrowth forms a narrow, slightly protruding transverse plate adherent to the anterior cloacal lip in ventral view, as visible in *C*. *gymnothoracis* n. sp. (Fig. 6D; [17, 22, 30, 44], present paper). The posterior cloacal lip may be conspicuously elevated in some *Cucullanus* spp. (e.g. *C*. *costaricensis* López-Caballero, Osorio-Sarabia & García-Prieto, 2009) or may be provided with a large, markedly elevating transverse outgrowth (*C*. *pedroi*, *C*. *austropacificus* n. sp., see Fig. 3C) [15, 44], present paper.

Consequently, when studying cucullanid species, as well as other nematodes, it is highly desirable to examine them by both LM and SEM. Of course, the use of molecular methods, if possible, is also important.

### Key to species of *Cucullanus* parasitic in anguilliform fishes (Anguilliformes)

1. Mouth markedly inclined dorsally. Cuticular lining of oesophastome consisting of complex set of thickened cuticularized pieces separated by sutures. Narrow cervical alae present. Male with ventral sucker and spicules 345–775 μm long. Parasitic mainly in Holarctic freshwater Salmonidae; in Europe also in *Anguilla anguilla* (Anguillidae) serving as postcyclic host .............. subgenus *Truttaedacnitis* ............................................ ***C*. *truttae***
  - Mouth slightly inclined dorsally. Cuticularized plates of oesophastome few in number and separated by simple Y-shaped suture. Cervical alae mostly absent, ventral sucker usually present .......... subgenus *Cucullanus* .................................................................................. 2
2. Ventral sucker absent. Spicules 636–924 μm long, length of gubernaculum 156–204 μm. Parasitic in *Muraenesox cinereus* (Muraenesocidae) in the East China Sea (off China) ............................................................................................................................. ***C*. *wangi***
  - Ventral sucker present ........................................................................................................ 3
3. Spicules shorter than 500 μm .............................................................................................. 4
  - Spicules at least 590 μm long .................................................................. ......................... 5
4. First pair of preanal papillae located short distance anterior to ventral sucker. Oesophastome elongate, longer than wide. Spicules 440 μm long, length of gubernaculum 90 μm. Parasitic in *Muraena robusta* (Muraenidae) off the Atlantic coast of Africa (Senegal) ............................................................................................... ***C*. *murenophidis***
  - First pair of preanal papillae at level of ventral sucker. Oesophastome approximately as long as wide. Spicules 430 μm long, length of gubernaculum 140 μm. Parasitic in *Muraenesox cinereus* (Muraenesocidae) off Japan ............................... ***C*. *muraenesocis***
5. First pair of preanal papillae at level of ventral sucker (near its anterior end). Spicules at most 800 μm long ............................................................................................................ 6
  - First pair of preanal papillae located short distance anterior to ventral sucker. Spicules usually longer than 800 μm .............................................................................................. 8
6. Oesophastome elongate, its length approximately one and half of its width. Length of spicules 800 μm. Parasitic in *Anguilla japonica* (Anguillidae) in Taiwan Strait (off China) ........................................................................................................................ ***C*. *anguillae***
  - Oesophastome approximately as long as wide. Spicules shorter than 800 μm ……..….. 7
7. Length of spicules 590–650 μm. Parasitic in *Anguilla anguilla* (Anguillidae) on the coast of the Red Sea (Gulf of Suez, Egypt) …………………….… ***C*. *egyptae*** [*species inquirenda*]
  - Length of spicules 637–760 μm. Parasitic in *Muraenichthys gymnopterus* (Ophichthidae) in the South China Sea (off China) ............................................................ ***C*. *hainanensis***
8. Deirids located somewhat posterior to nerve ring. Spicules 990–1,200 μm long. Excretory pore in region of deirids. Parasitic in *Gymnothorax pictus* and *G*. *javanicus* (Muraenidae) in the South Pacific (off Australia and French Polynesia) ..................... ***C*. *australiensis***
  - Deirids located at mid-length of oesophagus or anterior to oesophago-intestinal junction. Excretory pore slightly anterior, at level or posterior to end of oesophagus ................... 9
9. Oesophastome elongate, its length approximately one and half of its width .................... 10
  - Oesophastome approximately as long as wide ................................................................. 12
10. Oesophastome approximately as wide as posterior part of oesophagus. Spicules 600–1,260 μm long. Parasitic in *Conger conger* (Congridae) (type host) and, allegedly, *Muraena helena* (Muraenidae) off the Atlantic coast of Europe and Africa ......................... ***C*. *hians***
  - Oesophastome wider than posterior part of oesophagus ................................................. 11
11. Deirids located at posterior third of oesophagus. Median outgrowth adherent to inner rim of anterior cloacal lip as large as median precloacal papilla-like formation. Posterior cloacal lip with large, conspicuously elevated transverse outgrowth. Spicules 900–1,600 μm long, with conspicuously broad alae. Body length of male approximately 10–16 mm, that of gravid female 13–18 mm. Parasitic in *Conger orbignianus* (Congridae) off the Atlantic coast of Argentina .............................................................................. ***C*. *pedroi***
  - Deirids just anterior to oesophago-intestinal junction. Median outgrowth adherent to inner rim of anterior cloacal lip distinctly smaller than median precloacal papilla-like formation. Posterior cloacal lip without conspicuously elevated outgrowth. Spicules 668–1020 μm, spicular alae not conspicuously broad. Body length of male approximately 6–10 mm, that of gravid female 9–14 mm. Parasitic in *Anguilla marmorata* (type host) and *A*. cf. *obscura* (Anguillidae) in Polynesia and Melanesia (Futuna and Fiji Islands) ..................................................................................................................... ***C*. *oceaniensis***
12. Narrow cervical alae absent. Anterior and posterior cloacal lips without conspicuously elevated median outgrowths ……………………………………………………..……. 13
  - Narrow cervical alae present. Anterior and posterior cloacal lips each with conspicuously elevated median outgrowth. Gubernaculum 225–291 μm long. Length of spicules 1.30–1.65 mm. Body length of male approximately 16–26 mm, that of female 26–35 mm. Parasitic in *Conger cinereus* (Congridae) in the South Pacific (off New Caledonia) ……………………………....................................…………… ***C*. *austropacificus* n. sp.**
13. Deirids located at mid-length of oesophagus. Excretory pore slightly anterior to or at level of oesophago-intestinal junction. Phasmids of male situated between two last pairs of subventral postanal papillae. Gubernaculum 108–180 μm long. Length of spicules 980–1,600 μm. Body length of male approximately 7–18 mm, that of female 14–26 mm. Parasitic in *Conger myriaster* (Congridae) off Japan and Korea ................. ***C*. *robustus***
  - Deirids somewhat anterior to end of oesophagus. Excretory pore short distance posterior to oesophago-intestinal junction. Phasmids of male situated anterior to last two pairs of subventral postanal papillae. Gubernaculum 201 μm long. Length of spicules 1116 μm. Body length of male 15 mm. Parasitic in *Gymnothorax chilospilus* (Muraenidae) in the South Pacific (off New Caledonia) ……………………..…… ***C***. ***gymnothoracis* n. sp.**
